# Cost-effectiveness of stress CTP versus CTA in detecting obstructive CAD or in-stent restenosis in stented patients

**DOI:** 10.1007/s00330-020-07202-z

**Published:** 2020-09-03

**Authors:** S. H. Kim, J. Rübenthaler, D. Nörenberg, T. Huber, W. G. Kunz, W. H. Sommer, S. O. Schoenberg, S. Janssen, D. Overhoff, M. F. Froelich

**Affiliations:** 1 Department of Radiology, University Hospital, LMU Munich, Marchioninistr. 15, 81377 Munich, Germany; 2 Department of Radiology and Nuclear Medicine, University Medical Center Mannheim, Theodor-Kutzer-Ufer 1-3, 68167 Mannheim, Germany

**Keywords:** Cost-benefit analysis, Tomography, X-ray computed, Computed tomography angiography, Coronary artery disease, Stents

## Abstract

**Objectives:**

The aim of this retrospective study was to determine cost-effectiveness of stress myocardial CT perfusion (CTP), coronary CT angiography (CTA), and the combination of both in suspected obstructive coronary artery disease (CAD) or in-stent restenosis (ISR) in patients with previous coronary stent implantation.

**Methods:**

A decision model based on Markov simulations estimated lifetime costs and quality-adjusted life years (QALYs) associated with CTA, CTP, and CTA + CTP. Model input parameters were obtained from published literature. Probabilistic sensitivity analysis was performed to evaluate overall model uncertainty. A single-variable deterministic sensitivity analysis evaluated the sensitivity of the results to plausible variations in model inputs. Cost-effectiveness was assessed based on a cost-effectiveness threshold of $100,000 per QALY.

**Results:**

In the base-case scenario with willingness to pay of $100,000 per QALY, CTA resulted in total costs of $47,013.87 and an expected effectiveness of 6.84 QALYs, whereas CTP resulted in total costs of $46,758.83 with 6.93 QALYs. CTA + CTP reached costs of $47,455.63 with 6.85 QALYs. Therefore, strategies CTA and CTA + CTP were dominated by CTP in the base-case scenario. Deterministic sensitivity analysis demonstrated robustness of the model to variations of diagnostic efficacy parameters and costs in a broad range. CTP was cost-effective in the majority of iterations in the probabilistic sensitivity analysis as compared with CTA.

**Conclusions:**

CTP is cost-effective for the detection of obstructive CAD or ISR in patients with previous stenting and therefore should be considered a feasible approach in daily clinical practice.

**Key Points:**

• *CTP provides added diagnostic value in patients with previous coronary stents*.

• *CTP is a cost-effective method for the detection of obstructive CAD or ISR in patients with previous stenting*.

**Electronic supplementary material:**

The online version of this article (10.1007/s00330-020-07202-z) contains supplementary material, which is available to authorized users.

## Introduction

Until today, invasive fractional flow reserve (FFR) has been universally considered the reference standard in evaluating the hemodynamic relevance of obstructive coronary artery disease (CAD) [1, 2]. However, the role of cardiac imaging in evaluating CAD is steadily growing in importance [3], mainly owing to its non-invasive nature [4].

Coronary computed tomography angiography (CTA) is one of the most common cardiac imaging modalities and it is widely recognized for its diagnostic accuracy in the detection of CAD, especially in patients with a low pre-test probability for CAD [5, 6]. Yet, CTA is not recommended in patients with prior coronary stenting [7], primarily due to beam hardening artifacts originating from metallic stent struts [8–10] and the high atherosclerotic burden in non-stented segments which often results in the overestimation of CAD severity [11].

In recent years, stress myocardial computed tomography perfusion (CTP) has gained increasing recognition as an imaging method which combines both anatomical and functional assessment in a single modality. CTP has been repeatedly shown to improve diagnostic accuracy in the detection of obstructive CAD in general [12–21], and few studies have also demonstrated the diagnostic value of CTP in patients with previous coronary stents [22, 23].

Nonetheless, cost-effectiveness of CTP for the evaluation of obstructive CAD or in-stent restenosis (ISR) in stented patients has not been evaluated yet and thus remains scientifically uncertain. To further investigate the role of CTP in the management of stented patients with suspected obstructive CAD, we determined the relative costs and cost-effectiveness of CTP, CTA, and the combination of both.

## Materials and methods

### Model overview

**Fig. 1:**
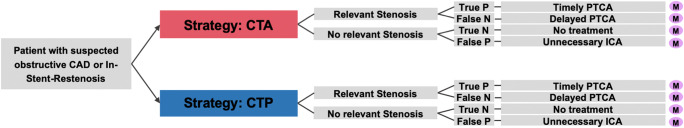
Model structure. Patients with previous stent implantation enter the model on admission for suspected obstructive CAD or ISR, receive diagnostic CT imaging and, depending on the result, may receive treatment

A decision model based on Markov simulations estimated lifetime costs and quality-adjusted life years (QALYs) for suspected obstructive CAD or ISR in patients with previous stent implantation, depending on the selected diagnostic imaging modality (Fig. 1). Within the simulation, costs of a timely percutaneous transluminal coronary angioplasty (PTCA) were applied if the diagnostic result was true positive. Costs of a delayed PTCA were applied in the case of false negative whereas a true negative did not entail any acute costs as no treatment was required. In case of a false positive, costs of an unnecessary invasive coronary angiography (ICA) without revascularization were applied.

For outcome analysis, a Markov transition state model including the states “alive without symptomatic stenosis requiring therapy,” “alive with symptomatic stenosis requiring therapy,” and “dead” was applied (Supplement 1).

### Input parameters

Model input parameters were derived from systematic review of recent literature (Table 1). The pre-test probability of a stenosis requiring PTCA in patients with clinical suspicion of ISR or CAD progression was set to 62.67% in accordance with the literature [22, 24].

**Table 1:** Model input parameters

	Estimate	Distribution	Source
Name			
Pre-test probability of stenosis requiring PTCA	62.67%	β	Andreini et al 2019
Expected value	65		Andreini et al 2019
Assumed willingness to pay/QALY	$100,000.00		
Discount rate	3.00%		
Diagnostic performances			
CT angiography (CTA) sensitivity	100.00%	β	Andreini et al 2019
CTA specificity	38.60%	β	Andreini et al 2019
CT perfusion (CTP) sensitivity	91.40%	β	Andreini et al 2019
CTP specificity	78.90%	β	Andreini et al 2019
Costs (acute)			
CTA	$397.87	γ	Medicare (CPT 75574)
CTP	$470.31	γ	Medicare (CPT 75574 + CPT 93015; based on SCCT coding guidelines [47])
Invasive coronary angiography (ICA) + FFR	$2810.00	γ	Medicare (CPT 93454)
Percutaneous transluminal coronary angioplasty (PTCA)	$4678.00	γ	Medicare (CPT 37246)
Delayed PTCA, additional hospitalization costs	$6081.40	γ	Assumption to be 1.3× as expensive
Costs (long term)			
Yearly costs with relevant stenosis	$7588.45	γ	Assumption to be 1.3× as expensive
Yearly costs without relevant stenosis	$5837.27	γ	Weintraub et al 2008
Utilities			
QOL with relevant stenosis	0.70	β	Weintraub et al 2008
QOL without relevant stenosis	0.75	β	Weintraub et al 2008
Death	0	β	
Transition probabilities			
Risk of new relevant stenosis	0.0264	β	Bønaa et al 2016
Risk of death with relevant stenosis	0.0230	β	Bittencourt et al 2014
Risk of death without relevant stenosis	0.0232	β	Bittencourt et al 2014
Risk of death for other causes	0.0126	β	US Life Tables 2015

#### Diagnostic accuracy parameters

The diagnostic accuracy measures were adopted from a prospective study by Andreini et al [22]. This study evaluated the diagnostic value of CTA, CTP, and CTA + CTP in 150 patients with previous stent implantation referred for ICA with clinical suspicion of ISR or CAD progression. Patients with previous myocardial infarction, contraindications to the administration of adenosine, impaired renal function, or a body mass index > 35 kg/m^2^ had been excluded. All enrolled patients were subjected to a rest coronary CTA, a static stress myocardial CTP, and an ICA with additional invasive FFR if indicated. Diagnostic accuracy of CTA, CTP, and CTA + CTP was assessed in stent-, territory-, and patient-based analyses. Further details on the study design, patient population, and CT scan protocols have been reported previously [22, 24]. Andreini et al concluded that CTP demonstrated significantly higher diagnostic rate (96% vs. 68%) and diagnostic accuracy (86.7% vs. 76.7%) as compared with coronary CTA [22].

Although few other studies with considerably smaller study populations have addressed the added diagnostic value of CTP in evaluating CAD in patients with previous stent implantation [23, 25], these were not comparable due to a widely differing diagnostic algorithm [23] or a missing per-patient analysis [25].

#### Utilities

Utilities were assigned to the different health states to adjust survival for quality of life and were expressed as quality-adjusted life years (QALYs) by multiplying the time period spent in a health state with its respective utility. In the Markov model, the quality of life of patients with a relevant stenosis was set to 0.70 as compared to 0.75 for patients without relevant stenosis. These values were based on published evidence [26], assuming that patients without relevant stenosis had a quality of life equal to that of patients with obstructive CAD 6 months after revascularization therapy.

#### Cost estimates

Based on a US healthcare perspective, costs of diagnostic procedures were extracted based on the specific current procedural terminology (CPT) codes for each modality (Table 1). In the event of a true positive, costs of a timely PTCA were applied. These costs were assumed to rise by a factor of 1.3 in case of a false negative to account for expenses from an extended hospital stay and additional diagnostic procedures. A false positive was assumed to entail costs of an ICA without revascularization. Yearly costs for a patient without relevant stenosis were based on estimates from previous literature [27] which were inflated to 2019 values on the basis of the Medical Care Component of the Consumer Price Index of the US Bureau of Labor Statistics [28]. A relevant stenosis was presumed to result in yearly costs 1.3 times higher than in patients without relevant stenosis.

#### Transition probabilities

The independent transition probabilities were derived from previous literature [29, 30]. Risk of new relevant stenosis was estimated to be equal to the yearly rate of revascularization after PTCA and stenting [30]. Risk of death with or without relevant stenosis signifies all-cause death per year in obstructive or non-obstructive CAD [29]. The age-specific risk of death was derived from the US Life Tables of the year 2015 as the largest source of epidemiological data [31].

### Analysis

#### Cost-effectiveness analysis

Lifetime costs and QALYs associated with the two diagnostic strategies were calculated by the simulation model. In accordance with published recommendations [32], all future healthcare costs and QALYs were discounted at an annual rate of 3.0%. Cost-effectiveness was assessed based on a willingness to pay (WTP) threshold of $100,000 per QALY based on a recent systematic review discussing medical cost-effectiveness thresholds [33]. The analysis was conducted from a US healthcare system perspective throughout a lifetime horizon with all costs calculated in 2019 USD. The model was created as a decision tree using dedicated decision analysis software (TreeAge Pro version 19.1.1, TreeAge Software, LLC). All analyses were performed in a total time frame of 10 years after initial diagnostic procedures.

#### Sensitivity analysis

To evaluate the sensitivity of the results to plausible variations in model inputs, a single-variable deterministic sensitivity analysis including diagnostic accuracies and costs for the respective variables was performed. Results are visualized as a tornado diagram of incremental net monetary benefit (INMB) at $100,000. INMB refers to the difference of a new strategy (CTP) and that of the reference (CTA) in net monetary benefits which is the monetary value of a strategy at a specific WTP [34]. A positive INMB implies superiority of the new strategy.

Probabilistic sensitivity analysis was performed to evaluate the overall model uncertainty. Monte Carlo simulations using 30,000 iterations were run to derive a cost-effectiveness scatter plot.

## Results

### Cost-effectiveness analysis

In the base-case scenario with WTP of $100,000 per QALY, CTA resulted in total costs of $47,013.87 and an expected effectiveness of 6.84 QALYs, whereas CTP resulted in total costs of $46,758.83 with 6.93 QALYs. CTA + CTP reached costs of $47,455.63 with 6.85 QALYs. Therefore, strategies CTA and CTA + CTP were dominated by CTP in the base-case scenario.

### Deterministic sensitivity analysis

#### Effect of diagnostic accuracy on model

**Fig. 2:**
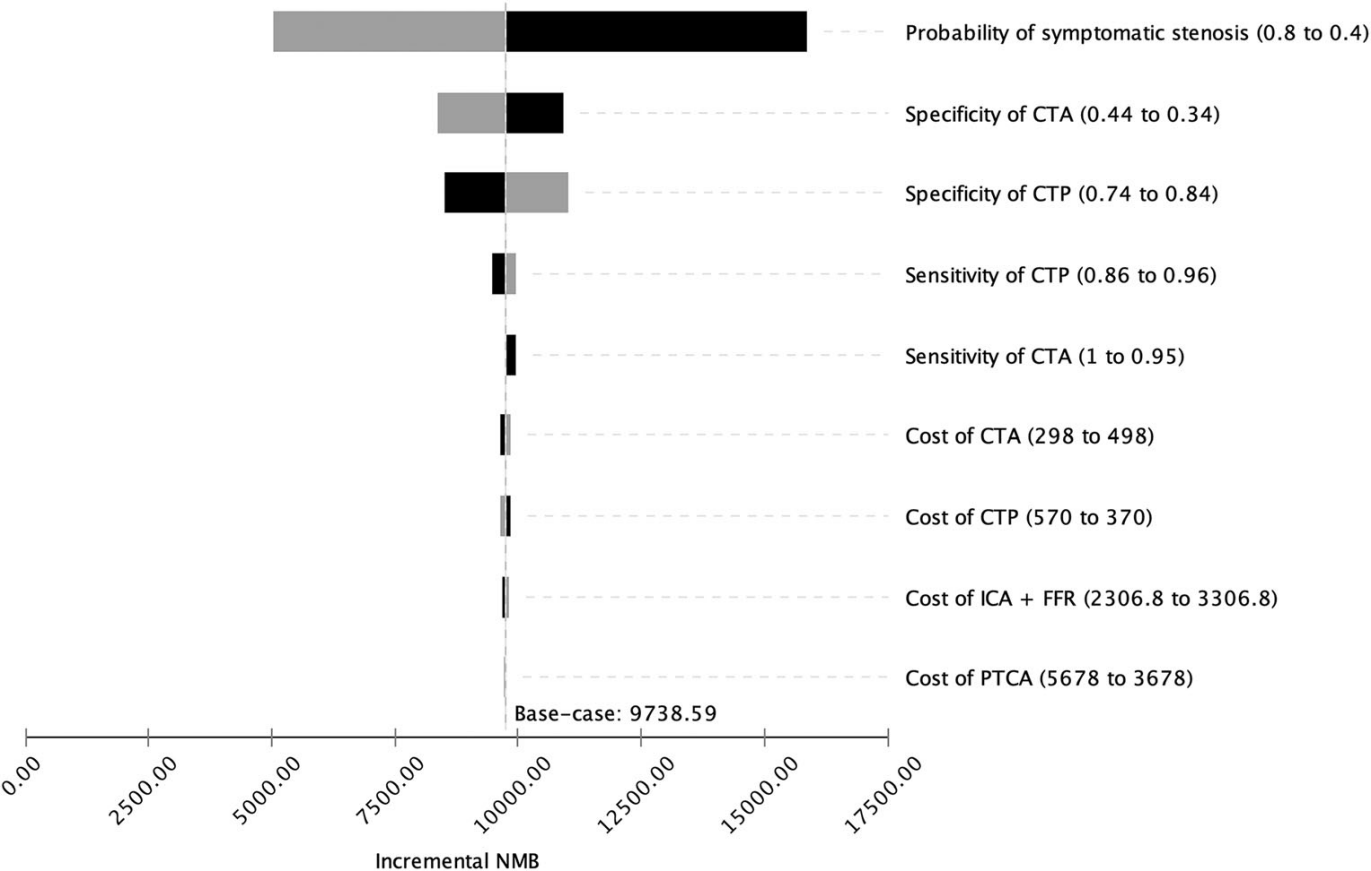
Tornado diagram. Deterministic one-way sensitivity analysis of input parameters. Incremental net monetary benefit (INMB) per patient for CTP compared with CTA is depicted based on a willingness to pay of $100,000/QALY. The plots show the INMB depending on several ranges of input parameters. For the ranges examined in the one-way sensitivity analysis, CTP results in a positive INMB

To evaluate the simulated model in detail, a one-way deterministic sensitivity analysis for the diagnostic accuracy measures (sensitivity and specificity) of CTA and CTP was performed. For both diagnostic strategies, sensitivity and specificity in a range of ± 5%, the assumed baseline values were analyzed. Within this range, the INMB maintained positive values and therefore CTP remained the cost-effective strategy (Fig. 2).

#### Effect of diagnostic and treatment costs on model

The potentially higher cost of CTP was taken into account within a broad range from $370 up to $570. Within these boundaries, CTP remained the dominant strategy (Fig. 2). The impact of cost variations for ICA in a range of ± $500 and for PTCA in a range of ± $1000 was evaluated. Within these value ranges, CTP remained the dominant strategy (Fig. 2). In sum, sensitivity analysis showed CTP to be the cost-effective alternative along a broad range of costs.

#### Effect of pre-test probability of symptomatic stenosis on model

The greatest uncertainty of the model lied in the pre-test probability of a symptomatic stenosis. Still, in a wide range or 40 to 80%, the incremental net monetary benefit (INMB) maintained positive values and therefore CTP remained the cost-effective strategy (Fig. 2).

### Probabilistic sensitivity analysis

**Fig. 3:**
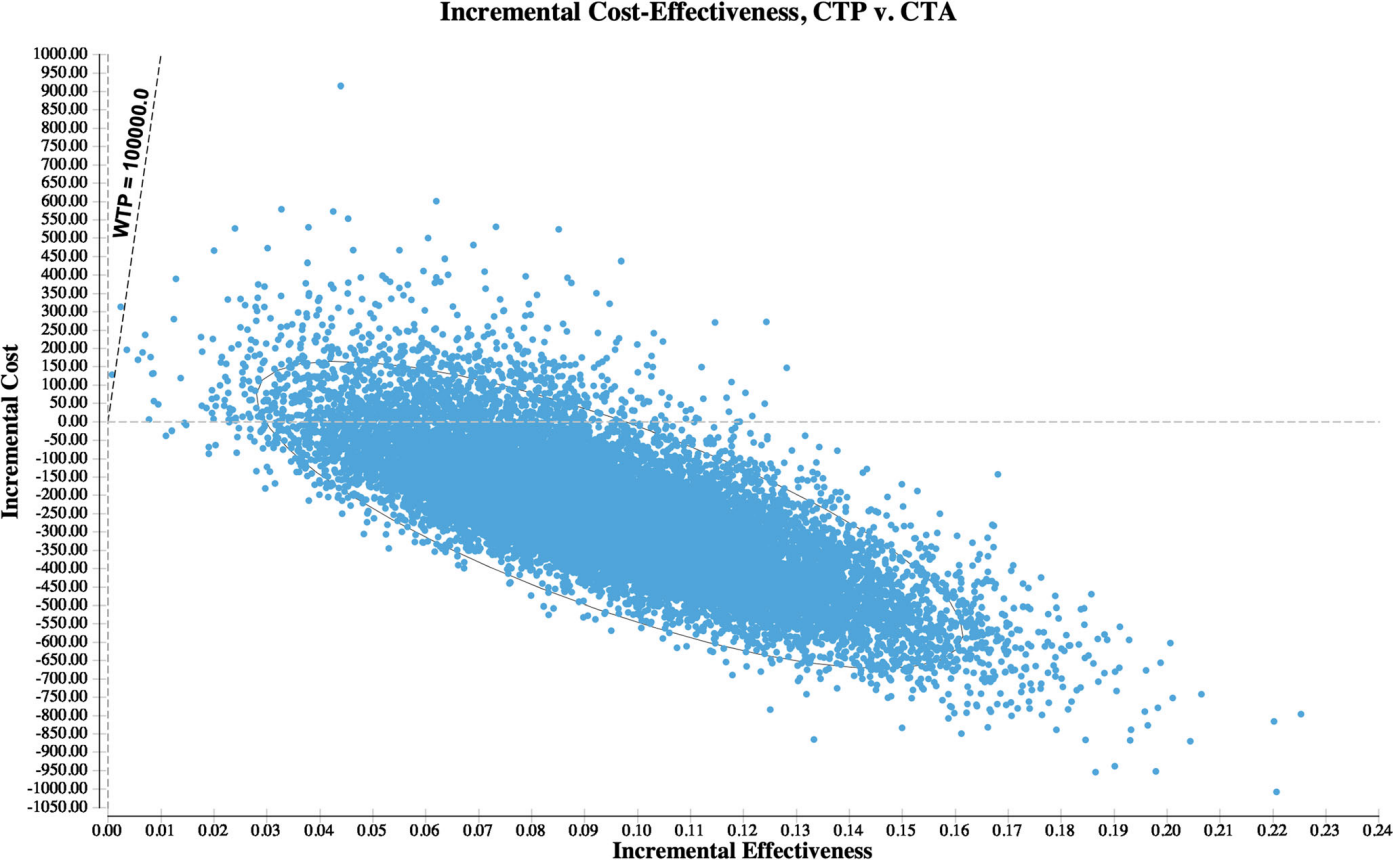
Incremental cost-effectiveness scatter plot. Results from probabilistic sensitivity analysis depicting incremental effectiveness and incremental costs of CTP when compared with CTA. Each point represents one simulation run. The dashed line represents the willingness to pay (WTP) of $100,000 per QALY. Simulation runs on the right side of this line are regarded as cost-effective

CTP was cost-effective in the majority of cases. At WTP of $100,000, the strategy was cost-effective in 99% of iterations (Fig. 3).

## Discussion

The aim of the present economic analysis was to evaluate cost-effectiveness of CTP for the diagnosis of obstructive CAD or ISR in stented patients. Our study demonstrated that CTP leads to greater QALYs and lower healthcare costs for stented patients with suspected obstructive CAD or ISR, as compared with CTA. Essentially, these findings can be attributed to the significantly lower specificity of CTA [22] which largely originates from susceptibility of CTA to movement and metal artifacts [8–10] as opposed to CTP which can appraise any coronary segment. Low specificity implies a high percentage of false positive results, eventually leading to a large number of costly, unnecessary ICAs. The results of the sensitivity analysis revealed robustness of the model to variations of diagnostic efficacy parameters, costs, and pre-test probability of a symptomatic stenosis in a broad range (Fig. 2).

In recent years, CTP has emerged as a promising cardiac imaging modality for the evaluation of hemodynamic significance of CAD [12–21]. Although the optimal diagnostic algorithm is a matter of ongoing debate, the most common regime is to perform coronary CTA first and add CTP only in equivocal or non-diagnostic cases, avoiding additional radiation in normal or insignificantly abnormal CTA findings [20, 21, 23]. Such a combined approach of CTA with CTP was shown to improve diagnostic accuracy and reduce referral rate for unnecessary ICA and revascularization [16, 19]. Importantly, the incremental value of additional CTP was demonstrated in patients across a wide spectrum of pre-test probabilities and coronary artery calcification [18]. The application of CTP is particularly interesting in the evaluation of patients with previous PCI in the light of the limited diagnostic accuracy of CTA in this patient subgroup [22–25].

Another modern technique that was shown to help avoid unnecessary ICA is FFR derived from CTA (FFR_CT_), either through computational fluid dynamic modeling or deep machine learning algorithms [35, 36]. However, as a post-processing approach derived from CTA data, FFR_CT_ is prone to the same artifacts limiting CTA evaluation. Therefore, the application of current FFR_CT_ techniques is limited to native vessels and the diagnostic accuracy of FFR_CT_ in patients with prior PCI or CABG remains to be explored [37].

It is important to note, however, that despite the growing evidence supporting the diagnostic potentials of CTP, even the latest guidelines do not explicitly recommend this modality, neither for the detection of CAD in general nor for stented patients [2, 38]. Until now, there is no consensus of the optimal scanning mode of CTP and the protocols may vary across institutions which hinders the structural implementation of this technique across a broad range of institutions [39]. Additionally, the use of CTP is associated with a higher radiation dose burden and an exact guideline for patient referral to this imaging modality should be drawn up in the future. Optimization of protocols to reduce radiation dose should be developed while maintaining image quality [40]. Iterative reconstructions will help in this context. Future studies should demonstrate the benefit of CTA and CTP in symptomatic patients with prior stent implantation or coronary bypass graft since these patient subgroups have been excluded by most previous studies, as highlighted by a recent meta-analysis by Hamon et al [16]. The demonstration of the cost-effectiveness of this modality is an important step in further establishment and clinical use of this imaging technique, especially in a health economic context where allocation of resources is determined not only by medical aspects but also by economic considerations.

### Study limitations

First, there is only very limited published data for the diagnostic accuracy measures of CTA and CTP in the detection of obstructive CAD in stented patients. Although a systematic meta-analysis would be more preferable, due to lack of such data, the current model was based on the largest applicable study available which included 150 patients [22]. Also, the differences in per-person diagnostic accuracy between CTP and CTA are larger in this study [22] compared with another study by Rief et al [23], which is why the results of the current simulation study may be exaggerated. Second, this study could not adequately evaluate the incremental value of CTA + CTP as a more likely clinical scenario. Although this strategy was included in the analysis, the results are not meaningful due to the decision algorithm used in the study by Andreini et al, which the diagnostic accuracy values were derived from [22]. The authors of this study classified all cases of discordant CTA and CTP findings as positive which led to a very low specificity of the combined approach (sensitivity: 100%, specificity: 42.9%). This resulted in a high false positive rate of 21.3% leading to a high number of costly, unnecessary ICAs within the simulation model. Accordingly, the strategy CTA + CTP was dominated by CTP alone in this cost-effectiveness analysis. The incremental value of CTA + CTP may be better evaluated if a different decision algorithm is used for the study design. For instance, the application of CTP only in non-evaluable or equivocal CTA findings may result in higher diagnostic accuracy and reduced costs. This approach may possibly render CTA + CTP cost-effective as compared with CTP alone.

Third, several other available cardiac imaging modalities were not included in the analysis. Alternative imaging methods for the detection of CAD that have been previously reported include stress cardiac magnetic resonance imaging [41, 42], stress echocardiography [43–45], CT-FFR [35, 36], SPECT [36, 42, 43], and PET [46]. Yet, the specific diagnostic accuracy of these methods in stented patients has not been adequately examined in previous studies, and hence a respective comparative cost-effectiveness analysis was not feasible.

Fourth, this cost-effectiveness analysis was performed based on US healthcare cost data and its findings cannot be immediately transferred to other healthcare systems. For instance, US healthcare services are typically more expensive compared with healthcare services in European countries.

Finally, varying levels of radiation exposure for CTA and CTP were not reflected in the calculation of lifetime QALYs. Nevertheless, the mean effective radiation dose for coronary CTA and stress CTP reported by Andreini et al [22] differed only marginally (1.87 mSv vs. 2.26 mSv for *K* = 0.014 mSv/mGy cm) and were therefore considered negligible for the purpose of the present analysis.

## Conclusion

CTP is cost-effective for the detection of obstructive CAD or ISR in patients with previous stenting and therefore should be considered a feasible approach in daily clinical practice.

## Electronic supplementary material

ESM 1 (DOCX 235 kb)

ESM 2 Supplement 3: Results of base-case scenario. (XLSX 9 kb)

ESM 3 Supplement 4: Results of deterministic sensitivity analysis. (XLSX 9 kb)

## Abbreviations

CAD: Coronary artery disease
CTA: Coronary computed tomography angiography
CTP: Adenosine-induced stress myocardial computed tomography perfusion
FFR: Fractional flow reserve
ICA: Invasive coronary angiography
ICER: Incremental cost-effectiveness ratios
INMB: Incremental net monetary benefit
ISR: In-stent restenosis
PTCA: Percutaneous transluminal coronary angioplasty
QALY: Quality-adjusted life year
WTP: Willingness to pay
