# Supplementary material for: Cost-effectiveness of stress CTP versus CTA in detecting obstructive CAD or in-stent restenosis in stented patients
Source: Eur Radiol. 2020 Sep 3;31(3):1443–50. doi: 10.1007/s00330-020-07202-z (PMC7880924; doi:10.1007/s00330-020-07202-z)
Supplement: Supplementary file 1 — (DOCX 235 kb) [file 330_2020_7202_MOESM1_ESM.docx]

***Supplement 1****: Markov model. Outcomes are modeled in three health states.*


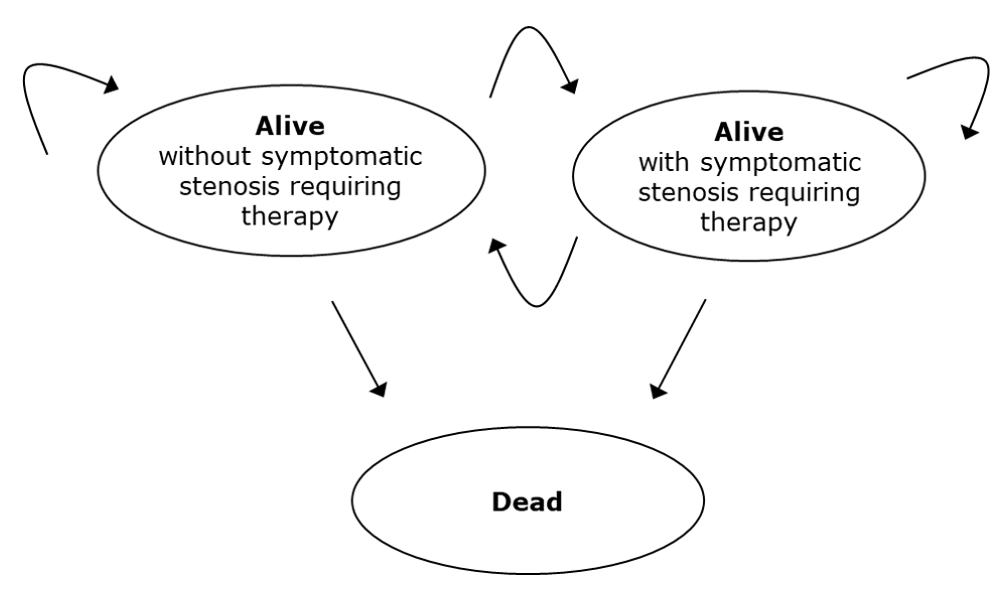


***Supplement 2:*** *Decision tree model.*

*
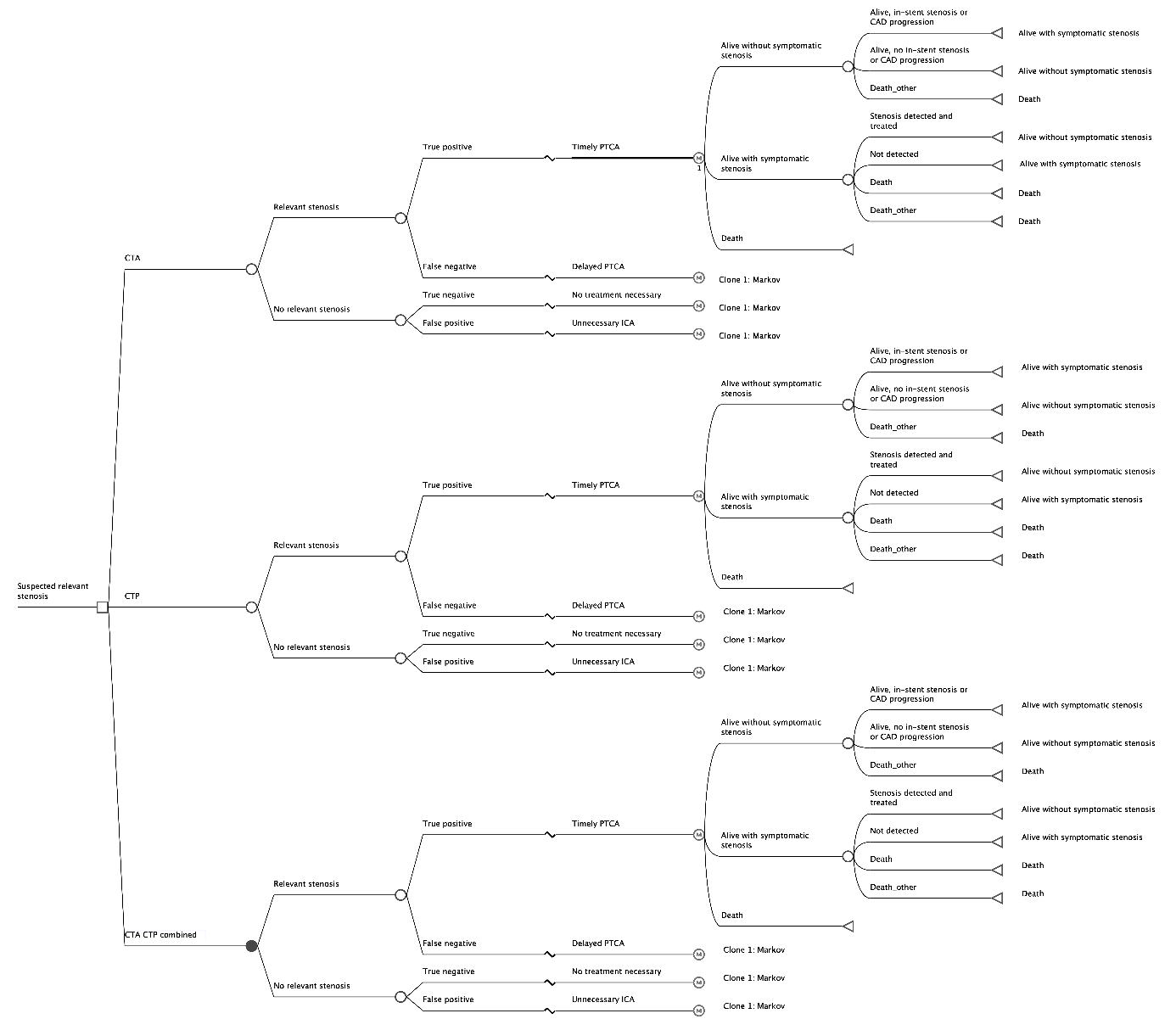
*

***Supplement 3:*** *Results of base-case scenario.*

***Supplement 4:*** *Results of deterministic sensitivity analysis.*
